# Unlocking Enhanced Efficacy of Aminoglycoside Antibiotics Against *Pseudomonas aeruginosa*

**DOI:** 10.1111/1751-7915.70174

**Published:** 2025-05-30

**Authors:** Patrick Ofori Tawiah, Sadia Sultana, Jan‐Ulrik Dahl

**Affiliations:** ^1^ School of Biological Sciences, Microbiology Illinois State University Normal Illinois USA

**Keywords:** adjuvants, aminoglycosides, antibiotics, metals, *Pseudomonas aeruginosa*

## Abstract

Infectious diseases continue to be a global health burden. Among the major human pathogens is the Gram‐negative bacterium *Pseudomonas aeruginosa*, which is particularly due to its wide range of drug resistance mechanisms. Aminoglycosides, which have long been used in treating pseudomonal infections, are increasingly undermined by resistance. This opinion article discusses the use and challenges of aminoglycosides against *P. aeruginosa* and highlights recent strategies that enhance aminoglycoside efficacy. These include combinational therapies, metabolic stimulants/adjuvants, silver, and more recently developed derivatives such as AGXX, many of which have been reported to potentiate the cytotoxic effects of aminoglycosides and re‐sensitise aminoglycoside‐resistant strains. While these findings pave the way for future therapies, the clinical relevance of many in vitro studies remains to be investigated.

## Graphical abstract

This opinion article reviews the use and challenges of aminoglycosides on the Gram‐negative pathogen *Pseudomonas aeruginosa* and highlights recent discoveries of adjuvants and/or other compounds that potentiate the activity of these antibiotics.

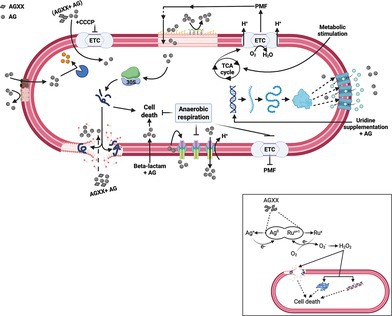

Over the past 5 years, much of the world's attention has been drawn to the coronavirus 2019 (COVID 19) pandemic and thus diverted from other significant health concerns. One such pressing global challenge in infectious disease control is the rise of antimicrobial resistance (AMR). AMR is caused by bacterial and fungal pathogens that have evolved resistance mechanisms towards an alarmingly high number of antimicrobials, including antibiotic treatments. The concern of AMR development is further amplified by the steep decline in the discovery of novel antibiotics over the last two decades. Infections caused by AMR are estimated to be directly responsible for over 1.27 million deaths in 2019 and contribute to an additional 3.68 million deaths worldwide (Murray et al. 2022). AMR infections have emerged in all antibiotic classes and could increase worldwide to 10 million deaths annually by 2050 (de Kraker et al. 2016) posing significant economic costs, which could exceed US$1 trillion in additional healthcare costs. AMR infections are particularly challenging for at‐risk populations such as immunocompromised patients (Church et al. 2006).

## 1 Resistance in Gram‐Negative Pathogens

**FIGURE 1:**
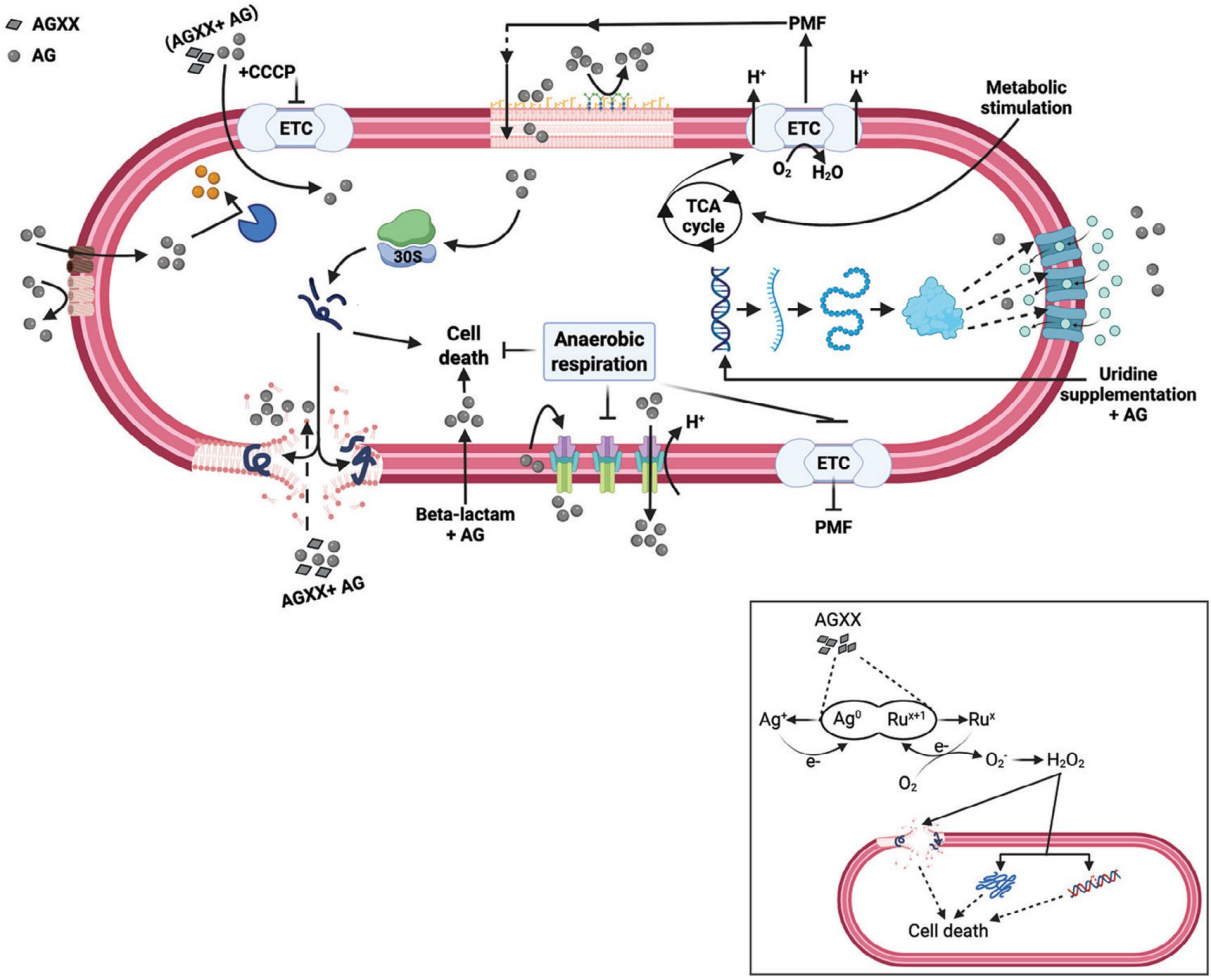
Unlocking the enhanced efficacy of aminoglycosides in *Pseudomonas aeruginosa*. Upon entry into the *P. aeruginosa* cell, which is mediated by the proton motive force, aminoglycosides (AGs, grey circles) target the 30S ribosomal subunit and impact protein translation. As a result, mistranslated proteins form toxic protein aggregates that either disrupt proteostasis and/or the membrane integrity, facilitating more antibiotic uptake, ultimately leading to cell death. However, bacteria have evolved several strategies to combat the bactericidal effects of AGs, including efflux pump expression, reduction of PMF due to a switch to anaerobic metabolism, and chemical modification of aminoglycosides. To circumvent this resistance, adjuvants could help to work synergistically with AGs. AG/adjuvant combinations have been shown to increase bacterial membrane permeability, block bacterial defence mechanisms, and cause an imbalance of the redox homeostasis in bacteria, resulting in AG uptake and improved bactericidal effects. For example, uridine stimulates the expression of sugar transporters, which enhances AG uptake. Likewise, the novel silver‐ruthenium complex AGXX, for which no resistance has been reported yet, exponentially increases uncontrolled AG uptake through membrane damage. Inset: Apart from its synergistic effects with AGs, AGXX generates reactive oxygen species through the redox cycling between silver (Ag) and ruthenium (Ru) when in contact with organic matter, which directly elicits macromolecular damage leading to bacterial cell death.

The resistance crisis is further exacerbated in Gram‐negative pathogens due to their outer membrane, an additional permeability barrier, and the extensive arsenal of drug resistance mechanisms these critters employ. The opportunistic pathogen *Pseudomonas aeruginosa* is one such difficult‐to‐treat Gram‐negative pathogen, and a common cause of acute (e.g., wounds, burns, eye infections, urinary tract infections) and chronic (e.g., diabetic ulcers, cystic fibrosis) infections. Characterised by a significantly larger genome size (5.5–7 Mbp) compared to other sequenced bacteria such as *Escherichia coli* (4.6 Mbp) or *Mycobacterium tuberculosis* (4.4 Mbp), *P. aeruginosa* encodes a disproportionally large number of regulatory enzymes important for the metabolism and transport of organic compounds (Stover et al. 2000; Klockgether et al. 2011; Pang et al. 2019). These enzymes provide the pathogen with great metabolic versatility and high adaptability to environmental changes, including exposure to antibiotics. *P. aeruginosa* has developed resistance to a variety of antibiotics from different classes, including aminoglycosides, quinolones and β‐lactams (Pang et al. 2019). The major resistance strategies of *P. aeruginosa* can be classified into intrinsic (i.e., low outer membrane permeability, presence of efflux pumps, and high production of antibiotic‐inactivating enzymes [Figure 1]), acquired (i.e., new genetic traits through either horizontal transfer of resistance genes or mutational changes) and adaptive mechanisms (i.e., formation of biofilms and tolerance towards multiple antibiotics), all of which contribute to prolonged or recurrent infections and require novel, alternative treatment strategies (Pang et al. 2019).

## 2 Aminoglycosides as Clinically Relevant Treatment

First discovered in the 1940s as a natural product in *Streptomyces griseus* (Krause et al. 2016), the class of aminoglycoside antibiotics is now comprised of compounds that were also isolated from other *Streptomyces* spp. (i.e., kanamycin, tobramycin, neomycin) or from *Micromonaspora* (i.e., gentamicin, sisomicin). In addition, semisynthetic derivatives have successfully been developed to combat AMR (i.e., amikacin) (Webster and Shepherd 2022). The core structure of aminoglycosides is characterised by amino sugars connected via glycosidic linkages to a dibasic aminocyclitol, which in most members of this antibiotic group is 2‐deoxystreptamine (Mingeot‐Leclercq et al. 1999; Krause et al. 2016). Aminoglycosides have long been used in antipseudomonal chemotherapy (Poole 2005), most commonly tobramycin (Fiel and Roesch 2022), amikacin (Lau et al. 1977) and gentamycin (Martin and Beveridge 1986), all of which are implicated in the treatment of a variety of *P. aeruginosa* infections, particularly pulmonary infections in cystic fibrosis patients. Due to their polycationic nature, aminoglycosides primarily inhibit the bacterial translational machinery by binding to the aminoacyl‐tRNA recognition site and interacting with the 16S ribosomal RNA, leading to mistranslation (Figure 1). While other protein synthesis inhibitors such as macrolides, chloramphenicol, or tetracyclines are bacteriostatic, aminoglycosides are unique in that they exhibit bactericidal effects. Several hypotheses explaining the cause of aminoglycoside‐mediated bacterial killing are currently under debate, including killing through uncontrolled antibiotic uptake induced by a positive feedback loop, membrane destabilisation through insertion of mistranslated proteins, dysregulation of the membrane potential and/or oxidative stress (Figure 1) (Webster and Shepherd 2022). Aminoglycoside uptake by bacteria occurs in three steps. First, ionic interactions occur between the polycationic antibiotic moieties and negatively charged components of the cell surface, including lipopolysaccharide (LPS) and outer membrane proteins (i.e., OmpF) (Martin and Beveridge 1986; Saika et al. 1998). The displacement of divalent cations from aminoglycoside binding sites destabilises and crosslinks the LPS components, leading to the disruption of outer membrane integrity in an energy‐independent way (Joshi et al. 2015). Subsequently, two energy‐dependent steps follow, which allow the polar drug, likely driven by an increased aerobic respiratory rate and elevated proton motive force, to cross the plasma membrane (Hancock 1981; Vanhoof et al. 1995). These discoveries link aminoglycoside activity to oxygen dependence and potentially explain why most anaerobic bacteria are resistant to this antibiotic class (Kohanski et al. 2008; Ramirez and Tolmasky 2010). Once aminoglycosides enter the bacterial cytosol, they interact with the 16S rRNA at the aminoacyl‐tRNA recognition site, stalling translation. Insertion of misfolded proteins into the plasma membrane was shown to cause the formation of irregular membrane channels that allow for increased aminoglycoside uptake and ultimately complete ribosome inhibition (Figure 1) (Davis et al. 1986; Kohanski et al. 2008). Likewise, ROS production caused by the hyperactivation of the electron transport chain has been proposed as a potential contributor to the killing mechanism of aminoglycosides (Davis et al. 1986; Kohanski et al. 2008; Dwyer et al. 2014), although conflicting observations have been reported (Liu and Imlay 2013).

## 3 Aminoglycoside Resistance Mechanisms

Resistance of *P. aeruginosa* to aminoglycosides has now been reported in all parts of the world, along with mechanistic details of resistance development (reviewed in Poole (2005)). While mutations in genes encoding ribosomal proteins have been associated with the resistance to some antibiotic classes (Unge et al. 1998; Justice et al. 1999), this mechanism appears to be irrelevant for aminoglycoside resistance. Likewise, methylation of 16S rRNA, which protects aminoglycoside‐producing organisms from the deleterious effects of the antibiotics, only plays a minor role in resistant *P. aeruginosa* (Thompson et al. 1985; Beauclerk and Cundliffe 1987). In contrast, resistant *P. aeruginosa* strains most commonly inactivate aminoglycosides through modifying enzymes, such as aminoglycoside phosphoryltransferases, aminoglycoside acetyltransferases, or aminoglycoside nucleotidyltransferases, which phosphorylate, acetylate, or adenylate the members of this antibiotic class, respectively (Poole 2005). Moreover, *P. aeruginosa* reduces the permeability of the outer membrane (Giamarellou and Antoniadou 2001; Wang et al. 2022) and activates the overexpression of efflux pump in response to aminoglycosides (Aires et al. 1999; Muller et al. 2011; Thacharodi and Lamont 2022), effectively reducing the intracellular drug concentration required for killing. Efflux genes generally exhibit low baseline expression in the absence of the selective pressure, and genetic changes resulting in loss‐ or gain‐of‐function phenotypes in their local regulators have frequently been identified as key factors driving overexpression and antibiotic resistance not only in clinical *P. aeruginosa* isolates but also in other bacterial species (Wu et al. 2024). The efflux activity of these pumps [reviewed in (Wu et al. 2024)] has even been reported to confer resistance to a broad range of antibiotics such as beta‐lactams, chloramphenicols and fluoroquinolones (Poole et al. 1993; Jamal et al. 2023) in both clinical and non‐clinical settings, such as bacteria‐plant interactions (Muller et al. 2015) and biocide resistance (Vargas et al. 2011).

Repeated aminoglycoside exposure can also prompt *P. aeruginosa* to develop adaptive resistance, resulting in previously susceptible populations becoming less responsive to the antibiotic over time (Barclay et al. 1992; Skiada et al. 2011). Long‐term evolutionary studies have also revealed single point mutations in the *fusA1* gene that are associated with increased aminoglycoside resistance (Feng et al. 2016; Yen and Papin 2017). Encoded by *fusA1*, the elongation factor EF‐G1A is involved in translocating the bacterial ribosome along mRNA during protein synthesis (Zhang et al. 2015). An amino acid substitution resulting from a single point mutation causes conformational changes in EF‐G1A that alter aminoglycoside binding to the 30S ribosomal subunit, thereby lowering the inhibitory effects of these antibiotics (Bolard et al. 2018). Mutations in *fusA1* have frequently been observed as a result of gentamicin (Rodriguez de Evgrafov et al. 2020) and tobramycin (Feng et al. 2016) treatments, as well as in clinical isolates of *P. aeruginosa* (Chung et al. 2012; Feliziani et al. 2014).

## 4 Unlocking the Enhanced Efficacy of Aminoglycosides in *P. aeruginosa*

A promising area of research has been directed towards the use of adjuvants and/or compounds that potentiate the efficacy of aminoglycosides by targeting metabolic processes or cellular networks (Figure 1). As a result, the synergistic effects of these combinations exponentially reduce the aminoglycoside concentrations required for efficient bacterial killing, which could reduce the risk and/or severity of side effects caused by the antibiotic and possibly override/delay resistance developments (Rosenberg et al. 2020). Synergistic effects have been reported between aminoglycosides and other antibiotics such as several beta‐lactam antibiotics (Giamarellou et al. 1984; Pankuch et al. 2011; Bilal et al. 2019), beta‐lactamase inhibitors (Agyeman et al. 2023), rifampicin (Poole et al. 2016) and macrolides (Tré‐Hardy et al. 2010). More recently, murepavadin (Martin‐Loeches et al. 2018), a member of a new antibiotic class effective against *P. aeruginosa* , has been shown to enhance the bactericidal efficacies of select aminoglycosides through increasing bacterial respiration and membrane potential that cause enhanced aminoglycoside uptake (Wei et al. 2024). The FDA‐approved antimicrobial triclosan also acts as an adjuvant and inhibits aminoglycoside‐induced adaptive resistance through depletion of the membrane potential, thereby eradicating *P. aeruginosa* biofilms (Maiden et al. 2018; Maiden and Waters 2020). Moreover, host‐derived antimicrobial peptides (Zhou and Peng 2013; Grassi et al. 2017), bacteriophages (Nicholls et al. 2023), antiviral drugs (Rezzoagli et al. 2020), heat‐shock (Lv et al. 2023), rapid freezing (Zhao et al. 2020), alkaline pH (Lebeaux et al. 2014), iron‐chelators (van Asbeck et al. 1983), inhibitors of the bacterial thioredoxin reductase (O'Loughlin et al. 2021), small molecule inhibitors such as halogenated indoles (Dou et al. 2023), and metabolites from both the host and *P. aeruginosa* sensitise the pathogen to aminoglycoside antibiotics (Barraud et al. 2013; Martins and Nguyen 2017; Crabbé et al. 2019). For instance, fumarate, a component of the tricarboxylic cycle, has been identified as a tobramycin‐specific potentiator that activates aerobic respiration and boosts the proton motive force in stationary phase *P. aeruginosa* , which improves killing significantly (Figure 1) (Meylan et al. 2017). Although direct evidence is lacking in *P. aeruginosa* , studies in *E. coli* and *Vibrio* species suggest that bacterial sugar transporters recognise the sugar component of aminoglycosides as a substrate, resulting in increased antibiotic uptake (Lang et al. 2023; Pierlé et al. 2023).

Likewise, silver and derivatives such as silver‐containing nanoparticles have been reported as potent inducers of aminoglycoside activity in *P. aeruginosa* and other bacteria (Kim et al. 2008; Morones‐Ramirez et al. 2013; Habash et al. 2017; Herisse et al. 2017; Barras et al. 2018; Dove et al. 2022). These potentially have meaningful clinical implications as silver derivatives have received increased attention in medical applications, for example, as antimicrobial surface‐coatings on catheters and implants aimed to protect from biofilm‐forming *P. aeruginosa* and reduce the risk of nosocomial infections (Lansdown 2010). Moreover, silver derivatives such as silverdene, despite being associated with allergic reactions to the sulfadiazine moiety, are used in topicals as a standard for treating and preventing *P. aeruginosa* infections in burn wounds (Fuller 2009). Recently, the novel antimicrobial AGXX has been reported to potentiate the cytotoxic activities of aminoglycosides and re‐sensitise aminoglycoside‐resistant *P. aeruginosa* strains, which is attributed to exacerbated intracellular ROS accumulation, increased membrane damage and elevated aminoglycoside uptake (Figure 1) (Donkor et al. 2023). The same study in *P. aeruginosa* also revealed that AGXX, which is comprised of the two transition metals silver (Ag) and ruthenium (Ru), is significantly more potent than classical silver and silverdene. The antimicrobial activity of AGXX is attributed to its specific coating composition and ability to generate reactive oxygen species (ROS) when in contact with organic matter. The elevated proteotoxic effects of AGXX alone and in combination with aminoglycosides reported in several independent studies are likely caused by AGXX‐generated ROS (Loi et al. 2018; Sultana et al. 2021; Donkor et al. 2023; Tawiah et al. 2025), as the antimicrobial effects of AGXX largely require oxygen availability (Donkor et al. 2023; Tawiah et al. 2025). However, Tawiah et al. (2025) reported that AGXX‐mediated DNA damage occurs ROS‐independently. Intriguingly, AGXX is non‐toxic to human cells, and no AGXX resistance has been reported yet, making it potentially well‐suited as a novel antimicrobial surface coating on medical devices.

## 5 Conclusion and Outlook

Despite the increasing demand for potent antipseudomonal drugs, monotherapeutic treatments may no longer be the best practice due to the rise in antimicrobial resistance. Many compounds synergize the activities of conventional antibiotics such as aminoglycosides, potentially revamping their use even in difficult‐to‐treat *P. aeruginosa* biofilms, persisters, or aminoglycoside‐resistant cells. However, several open questions remain, including whether and to what extent these reported synergies are relevant in vivo given that several host‐derived factors (i.e., oxygen availability, polyamines, iron limitation and aminoglycoside‐binding proteins) may severely impact the outcome of the synergy. Another concern, raised by O'Toole (2024), refers to the experimental conditions in which most studies were conducted. The ability of *P. aeruginosa* to co‐exist with other pathogens has been well accepted over the last decade, with significant impact on infection severity and persistence (Biswas and Götz 2021; Fischer et al. 2021; Bernardy et al. 2022; Shah et al. 2024). A recent in vitro co‐incubation study with *Staphylococcus aureus* identified mutations in *P. aeruginosa* LPS biosynthesis genes that increased *P. aeruginosa* ‐mediated killing of *S. aureus* but also enhanced the resistance of the Gram‐negative pathogen to beta‐lactam antibiotics (Tognon et al. 2017). Likewise, interactions of *P. aeruginosa* with other microorganisms alter their sensitivity to aminoglycosides (DeLeon et al. 2014; Jean‐Pierre et al. 2023).

## Author Contributions

**Patrick Ofori Tawiah:** writing – original draft. **Sadia Sultana:** writing – review and editing, visualization. **Jan‐Ulrik Dahl:** writing – review and editing, supervision, funding acquisition.

## Conflicts of Interest

The authors declare no conflicts of interest.

## Data Availability

Data sharing not applicable to this article as no datasets were generated or analysed during the current study.
